# Informing Streetscape Design with Citizen Perceptions of Safety and Place: An Immersive Virtual Environment E-Participation Method

**DOI:** 10.3390/ijerph20021341

**Published:** 2023-01-11

**Authors:** Marcus White, Nano Langenheim, Tianyi Yang, Jeni Paay

**Affiliations:** 1 Centre for Design Innovation, Swinburne University of Technology, Hawthorn, VIC 3122, Australia; 2 Melbourne School of Design, The University of Melbourne, Parkville, VIC 3010, Australia

**Keywords:** immersive virtual environments, e-participation, streetscape design, safe system treatments, road safety, place making, citizen perceptions, 4D modelling, 4D analysis

## Abstract

As our cities grow, it is important to develop policies and streetscape designs that provide pedestrians with safe comfortable walking conditions and acknowledge the challenges involved in making urban places feel liveable and safe while understanding the critical role of streets around busy destinations. To understand these challenges at a nuanced, human level, new methods of citizen engagement are needed. This paper outlines the development and application of a new citizen perception collection method, using immersive virtual environments (IVE), coupled with an interactive emoji affective activation-pleasure grid and digital slider elements, embedded within an online e-participation survey to quantify, and rank the impact of individual (single-variable) urban design elements and safe system treatments on pedestrians’ perceptions of safety and place. The results demonstrate the effectiveness of this method for providing detailed, interrogable, scalable citizen perception data of a variety of urban street design elements and safe system treatments, which allows a statistical analysis of responses and prioritization of the most effective pedestrian-oriented interventions for maintaining or enhancing street vibrancy and liveability. Our IVE e-participation approach is an important contribution to forming a better understanding of streetscapes and provides a valuable method for urban designers and transport planners to prioritise different streetscape place and safety approaches.

## 1. Introduction

### 1.1. Safety and Place

As Australian urban populations expand and cities adjust to accommodate higher densities, increased pressure is placed on already contested space within streets. To ensure these growing cities are vibrant, liveable, safe and promote healthy, sustainable transport choices for people, it is critical to develop policies and design streets that provide pedestrians with comfortable walking conditions to major destinations such as public transport, shops, healthy food, parks and recreation facilities [1]. As outlined in the Future Transport Strategy 2056, successful streets and roads meet the demands of both transport movement and place-making [2]. The Road Safety Plan 2021 identifies liveable and safe urban communities as priority areas to progress towards NSW road safety goals [3]. This plan acknowledges the challenges involved in keeping urban places liveable and safe and emphasises the important role those vibrant streets around busy destinations such as shopping centres, entertainment precincts and education facilities play in supporting and encouraging the movement of people, goods and services.

Vibrant streets are complex environments with many multisensory, interdependent variables such as traffic sound and speed, vegetation and trees, cycle lane and parking configurations, crossing types, footpath widths and shop diversity [4,5]. Each of these variables can be difficult to isolate in real-world environments, but each has a nuanced impact on human perception. Thus, a new, more accurate method of citizen engagement and collection of preference and perception data (both revealed and stated) is warranted, to improve our understanding, prioritisation and decision making around the most crucial aspects of urban quality.

### 1.2. Understanding Citizen Perceptions of Streetscapes

#### 1.2.1. Visual Representation

Pedestrian streetscape perceptions are affected by both static and dynamic variables. For example, a static spatial variable such as street width or the number of lanes can affect the perceived ease of making crossings, while dynamic visual and auditory variables associated with the time of day, speed, proximity to, and volume of adjacent vehicular traffic can have a significant impact on the perceived safety or convenience of making that crossing. While the use of images, either 2D photographs, photomontage or graphic illustration stimuli without sound have been found to have a high level of efficacy for eliciting preference data for static aspects of street configuration, studies aimed at understanding the impact of multisensory dynamic traffic conditions, require more complex stimuli and methods of data collection [6].

Even for static variables, there are limitations to using photographic stimuli for preference studies. Not only is photography of spatially comparable conditions, from equivalent locations with similar levels of maintenance, health, species, age and spacing of street trees, crowd sizes and their cultural background, gender and age, shop types and architectural variation required, but the quality of the imagery and camera used can also confound results, impacted by light conditions, the time of day, season and weather [7]. Understanding the impact of change in an individual variable using this method to generate visual stimuli, would require sourcing street environments with only a single difference, and then photographing or recording these environments under similar light, traffic and maintenance conditions [8,9]. These issues can be somewhat nullified through the use of the photomontage technique, where still images are modified to reflect a single variable change or through use of more abstract forms of representation, however there are accuracy and adaptability limitations to this method. Ultimately pedestrians do not experience streets from a fixed-framed perspective. People look around when they occupy a street.

Geographic Information Systems (GIS) data analysis-based methods for assessing the impact of measures of urban street green space and streetscape spatial proportions on rates of walking and perceptions of safety have also been developed [10,11]. However, as identified by Sakar, these methods have not accounted for the impact of changes in traffic parameters and thus cannot be used to understand causality. In addition, as outlined in the study by Harvey, measures were limited to the vertical spatial configuration of streetscapes rather than horizontal aspects such as lane widths, bike lanes and traffic proximity.

Overcoming some of the issues associated with still image visual assessment stimuli and GIS based methods, other studies have used a selection of paired video samples of existing streetscapes [4]. This method, while it is more suitable for understanding the impact of traffic movement on perceptions, also suffers from similar difficulties to those that arise in the selection of still photographic studies, with an added complication of sound variation. Nor is it easy for people to separate lived experiences of specific locations from their physical characteristics. Memories of specific events in specific locations can trigger psychological and physiological responses untethered to the physical proportions and conditions of the space in question.

#### 1.2.2. Immersive Virtual Environments (IVEs)

Virtual 3D environment models can overcome many of these problems more effectively than the previously-described methods as confounding factors can be minimised, individual variables can be controlled for, lived experiences and memory biases can be avoided, and movement and sound can be simulated in addition to visual and spatial aspects. While virtual environments must address the concept of embodiment (or how real it feels to exist in the simulated space) and levels of realism, virtual environments have been found to allow for the controlled adjustment of dynamic visual and auditory variables through movement, light and sound simulation [12,13].

Over the past three decades, virtual reality (VR) and immersive virtual environments (IVE) have been increasingly used to enable spatial understanding. These include IVEs viewed using enclosures with multi-directional projections such as Cave Automatic Virtual Environments (better known by the acronym CAVE) [14], interactive desktop applications, and through head mounted displays (HMDs) [15]. Over the last decade high-quality HMDs for consumers such as Oculus Rift and HTC Vive, that are significantly cheaper than the CAVEs and HMDs from the 1990s which cost in the tens of thousands of dollars have made these options more accessible, though still spatially bound to a single location.

An even cheaper, and more accessible approach is to use smartphones to achieve IVEs. By using a smart phone’s inbuilt gyroscopic sensor, it is possible to display a virtual environment in such a way as to allow the viewer to look around by simply moving the device. The user can download IVE three-dimensional models to the phone and interact with the environment in a similar intuitive way to the higher-end HMDs. They are, however, limited by the computational power of their specific smart device, meaning that an older or lower performance phone may be limited to very basic or abstracted geometry, material and light-rendering quality. A solution to this limitation is using 4-dimensional (3D plus time) animated computer graphics that allow IVEs to be pre-rendered as equirectangular projection videos. These 360-degree videos (also known as immersive videos or spherical videos) are rendered with metadata that informs the smartphone to project the video spherically and allow for interaction through the movement of the device using its gyroscope to track movement and move the user’s view on the virtual environment in response. Though not commonly known, this capability has been available on all Android and iPhone devices for several years and was even built into Google’s YouTube app [16]. Though this approach is not necessarily the highest-fidelity method for IVEs, the ubiquitous nature of this technology makes it ideal for high reach or crowdsourced research into user perceptions of spaces such as streets, as almost everyone already has the technology to interact with the IVEs in their pocket.

#### 1.2.3. 360-Degree Auditory Cues in Immersive Virtual Environments

Sound is an important aspect in supporting the feeling of presence and 'being there' in virtual environments [17]. Until recently, the study of presence in interactive virtual reality has been dominated by a focus on visual stimuli. While vision is regarded as dominant for spatial localisation and human experience [18], according to Larsson et al. [19], the auditory modality possesses unique features that may make it a deciding factor in achieving a full sense of presence. In the real world, our ears are 'always open', hence auditory perception cannot be turned off. Additionally, while the visual perception which supports our sense of the surrounding environment is inherently directional, as determined by our field of view, auditory perception is omni-directional. Spatial sound rendering can ‘create an impression of a sound environment surrounding a listener in 3D space, thus simulating auditory reality’ [19]. In addition to providing information about the environment beyond our field of view, sound can also influence perception of visible and tangible events and objects in the virtual environment [17]. Auditory cues can help establish direction and visual cues for navigation in virtual environments [20] while visual feedback helps the user interpret the sensory cues [21].

### 1.3. Aim

The aim of this study was to develop and test an immersive e-participation method using accurate, sound enhanced, single variable immersive virtual environments (IVE), to gain insights into citizen perceptions of design and safe system treatments on vibrant streets. This paper focuses on the overall development of the e-participation method. The detailed analysis of the survey results is beyond the scope of this method-focused paper and will be explored in detail in a forthcoming publication.

## 2. Method

To address this aim, in collaboration with the major Transport Authority for the state of New South Wales, Australia (TfNSW), we developed and deployed an interactive, immersive, online survey aimed at gathering affective, revealed and stated preferences for a series of streetscape designs. This process involved five key steps. Firstly, the identification of specific variables for streetscapes to be tested; secondly, construction of an animated digital 3D street model; thirdly, building an online survey with the embedded environments with demographic questions, revealed preference (emotional affect sliders and grid) and stated preference (ranking) questions; fourthly, recruiting participants; and finally, the statistical analysis of the results.

### 2.1. Identification of Specific Variables—Choosing Pedestrian-Oriented Place-Making and Safe System Treatments

**Figure 1:**
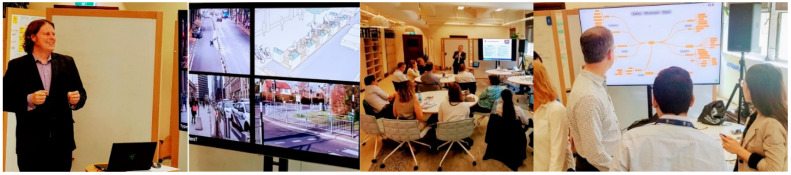
Photographs taken during the two-day stakeholder workshop held at the Future Transport Digital Accelerator.

For the study, an agreed set of safety and place variables for the investigation were scoped through two workshops run by the research team with TfNSW. Stakeholders input was sought in the workshops from a range of government groups with experience in road safety, safe systems, road network strategy and analysis, network planning transport, urban design and planning, customer (user) experience and design, economics, strategic policy and development, as well as design of public space [Figure 1].

**Figure 2:**
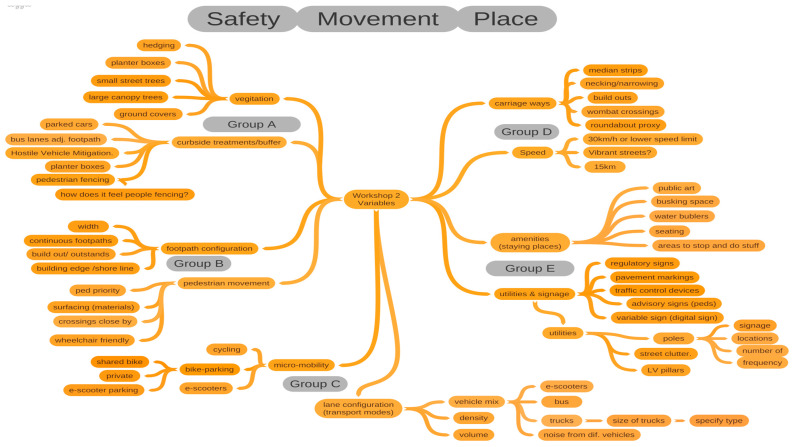
Collaborative mind map showing the ‘long-list’ of variables produced during the first workshop.

At the conclusion of the first workshop, stakeholders contributed to the production of a variables ‘long-list’ [see Figure 2] with input captured in an interactive collaborative mind-map tool (Coggle™). On the second day of the workshop, stakeholders explored, reviewed, sorted, and prioritized the long-list to develop a final short-list of high-priority, safety and place streetscape variables as the focus of the study.

The research team then rationalised these high-priority scenarios based on comparability, modelling feasibility and ability to achieve quantifiable results across a remote participant pool, accessing the survey on a wide variety of devices in uncontrolled environmental settings. This process saw scenarios involving lighting treatments excluded due to the inability to control for remote access settings such as weather, time of day, participant screen brightness or size and if the participant was in indoor or outdoor settings while completing the survey. In total, one ‘baseline’ of a ‘vibrant street’ and 12 priority scenario variations were chosen for inclusion in the study [Table 1]. Streetscape variations included one speed configuration, four barrier separation configurations, three distance separation configurations and four crossing configurations.

### 2.2. Construction of Animated Digital 3D Parametric Street Model

The team then developed a flexible digital 3D street scenario model, that could be rendered as an IVE with immersive spatial 360-degree audio as visual stimuli for eliciting participant responses. The model was constructed to allow controlled, isolated variable adjustments through parameter modification and layer control, i.e., bike lanes, vegetation buffers and barrier fence elements could be turned on or off, and animated vehicles incorporated speed and sound controls, allowing production of a consistent series of IVEs with specific, precise differences only in level of noise intensity, traffic speed, traffic distance, traffic separation from the footpath and crossing detail.

#### 2.2.1. 3D Digital Model Development

**Figure 8:**
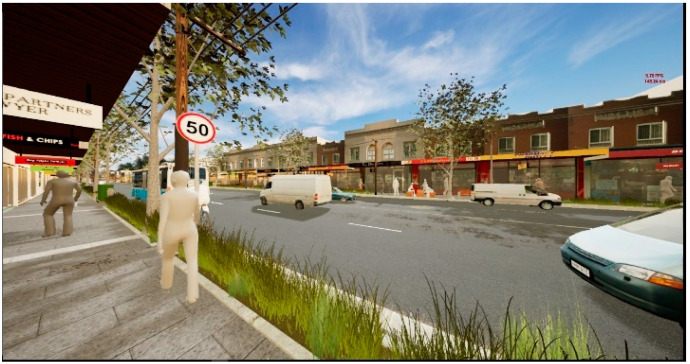
Screengrab of perspective view in game engine immersive virtual environment showing the ‘Ground vegetation buffer’ scenario.

The virtual environment geometry and textures were generated in 3D modelling and animation software. Buildings and shop fronts, roads, footpaths and street furniture assets were assembled as procedural objects, while the base streetscape geometry was parametrically modelled to maximise efficiency using constrained polygon counts and compressed textures to allow for rapid rendering. The base geometry was accurately modelled using dimensions and arrangements of a ‘typical’ twenty metres wide ‘main street’ with detailing informed by the relevant Technical Direction: For traffic and transport practitioners documents by TfNSW [22], including bike lanes treatments, crossings, footpaths, parking, signage and line-markings. The base geometry, driven by street centre line data, was a highly adaptive system that could rapidly be applied to a larger area or more complicated street configuration if required. We modelled custom vegetation for inclusion in the scenarios using water sensitive urban design (WSUD) plant types, and typical street trees commonly used in NSW streetscapes: *Dianella* sp., *Lomandra* sp., *Dwarf Bottlebrush*, *Clive* asp., *Juniper* sp., *Trachelospemum* sp. [Figure 8]. We scripted the vertices of the tree leaves and ground-level vegetation meshes so they could be randomly moved by a small offset amount to simulate a gentle breeze effect.

The geometry was textured with a mix of photographs, patterns and single colour elements to provide a sense of materiality and enhance the reading of spatial depth, while also minimising extraneous detail where it was not required, so the scenario variations could be clearly understood [23,24,25]. We added generic signage to add realism without specific cultural associations (not using real shop/restaurant names), so that the scenarios felt familiar but would not evoke specific place-based memories.

We added animated vehicles, trucks, buses, and motorcycles, at a volume and mix based on averages taken from the Daily Count Summary—Burwood Road and Deane Street, Burwood (data provided by TfNSW). The traffic volume was maintained in each of the different scenarios as much as possible, though minor variations in volumes occurred in some scenarios due to different lane configurations. To enhance the level of realism of the IVE experience, we used a variety of geometries for each type of vehicle in the simulation, including eight models of light vehicle, two models of truck, two models of bus and two models of motorbike. An artificial intelligence calculation system within the 3D modelling and animation software was used to simulate vehicle movement. Except for the ‘Reduced speed’ scenario (30 km/h), the speed limit for all vehicles was set to 50 km/h.

The crowd of animated pedestrians was simulated based on averages also taken from the Daily Count Summary—Burwood Road and Deane Street, Burwood. To reduce the ‘uncanny valley’ effect [26], and to reduce distraction, we textured their appearance in greyscale with different body types, giving an indication of a diverse population without providing specifics of skin colour, race or age. Additionally, to enhance the experience of immersion, we animated the crowd with a variety of behaviours, including walking, chatting, gesturing, talking on the phone, people with mobility impairment (using wheelchairs), and parents with baby strollers. This agent-based simulation of pedestrian and cyclist movement was programmed and filtered with behaviours driven by interaction ‘events’ between distinct entities. In other words, these simulated pedestrians and cyclists could avoid each other and street assets, and follow the traffic rule settings in different scenarios, for example, waiting for the traffic lights to cross the street.

#### 2.2.2. Game Engine Preparation

**Figure 9:**
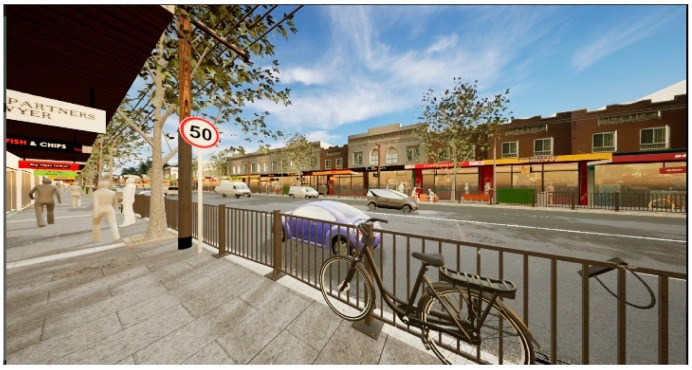
Screengrab of perspective view in game engine immersive virtual environment showing the ‘Barrier fence’ scenario.

Each complete scenario model was then transferred into the game development engine set to ‘virtual reality project’ allowing real-time control. Within the game engine, we optimised lighting and shadows, added dynamic simulation of trees and plants, programmed the traffic light cycles and added the audio. We used a High Dynamic Range Image (HDRI) background skydome to simulate a daylight-enriched quality of the materials and enhanced the reading of depth in the scenes and enhance the participants’ immersive experience. Lighting settings were kept consistent in all scenarios. The game engine ‘levels’ were adopted for streetscape scenario simulation management to handle complicated pedestrian simulation, cyclist simulation and vehicles simulation from the 3D modelling software. This approach guaranteed frame and camera setting synchronisation in the video outputs [Figure 8 and Figure 9].

In the IVE models, all sounds were set to ‘spatial audio’, which involved the manipulation of audio signals, so they mimicked acoustic behaviour of the real world. The traffic lights and crossing lights were programmed and synchronised with sound effects in the game engine. We set up vehicle engine noise based on typical RPM (engine revolution per minute) and related gear changing sound and logarithmic sound decay and intensity levels (DBa) from Vehicle noise levels for VR project with TfNSW Research Hub internal report provided by TfNSW. We simulated vehicle noise for the four key vehicle types at two speeds, 50 km/h and 30 km/h.

We regarded all simulated vehicles as agents in the game engine. We monitored the speed and acceleration of the agents, programmed and analysed the data by the scripts we built in the gameplay scripting system, constructed the relationship between speed, and acceleration of the agents and RPM-related engine sounds with correspondent pitch and volume. In this way, in the game engine simulation, the corresponding soundtracks of each category of vehicle would be attached to the geometry of the vehicle and played at appropriate sound power levels at the vehicle location while in free-flowing traffic. We added horn sound effects at the traffic congestion point to improve the realism of the scenarios. We also added four channels of ambient background city sound for each of the scenarios from recordings of the similar scaled ‘main street’—Glenferrie Road in Hawthorn, Victoria.

While we carefully modelled to control the quality and balance of sounds to provide as realistic an environment as possible, we did not have control over participant’s device hardware, headphone types, volume settings and video players, or the physical user environment of participants. To manage this limitation, we included instructions encouraging participants to use headphones and to adjust their computer or smart device’s audio settings to a comfortable volume that ‘feels realistic’.

#### 2.2.3. 360-Degree Video and Binaural Audio Output

While we initially envisaged conducting this study with citizens in person using virtual reality in head mounted displays, due to the impact of COVID19, it was not safe or feasible to conduct the experiential aspect of the streetscape scenarios this way. Instead, we adjusted the study to be fully online, and utilised an immersive 360-degree video approach.

In the game engine, we programmed and set up a panorama camera that constructed the scene with two lenses, to represent the virtual left and right eyes in the game engine scenario. We then projected the view captured by the lenses onto a 2:1 canvas in equirectangular form. The rendered scenes were exported as a series of equirectangular projections (flattened globe) image sequences which are 8K UHD 7680 × 3840 pixels per frame and 25 frames per second.

To export multi-directional sound (spatial audio), we recorded two channels of the audio at the point of the camera location while facing towards the street. We then recorded another two channels of the audio after horizontally rotating 90 degrees. The spatial audio was combined and rendered with the 8K UHD equirectangular image sequences in the video editing software and injected with specific 360-degree metadata. The specific metadata was embedded with the videos to help the video player platform (YouTube) to recognise and process 360-degree videos and spatial audio. We then uploaded 360-degree videos with spatial audio of thirteen scenarios to the YouTube video-sharing platform.

### 2.3. Online Survey—Testing Perceptions of Street Types with Varied Parameters

We developed the interactive survey using Qualtrics™ software to include these 360-degree IVE scenario models to be suitable for participants using either desktop/laptop (with a suitable web browser) or smart device (mobile smartphone or tablet). In the survey, we presented participants with nine different streetscape design scenarios, each with slightly different road configuration, crossing type, footpath or landscape elements. Though different streetscape designs involved many complex and interlinked variables, where possible, we minimised the number of variable changes in each scenario. We started with the ‘Baseline’ ‘typical main street’ scenario, (standard main street with two traffic lanes each way with 50 km/h speed limit), and then modified this street to represent the different streetscape treatments keeping all other variables the same, or as similar as possible given the requirements of the scenario. The intention of restricting variable changes was to allow for a variation on a discrete choice experimentation, a processed used to explore consumer decision-making in marketing and psychology [27].

The online survey consisted of five main sections. Firstly, an introduction and informed consent section, secondly a series of demographics questions, thirdly the scenario introduction and instructions, fourthly the randomised scenarios with associated safety and place questions, and finally a series of post-scenario questions.

#### 2.3.1. Introduction, Explanatory Statement and Informed Consent

As part of the study’s Ethics Committee approval conditions, the survey participants were first greeted with a project introduction and invited to read the full project details in the official Explanatory Statement. They were then asked to agree to the Informed Consent on the first question of the survey.

#### 2.3.2. Demographic Questions

The next set of questions invited participants to share demographic details, including age group, gender, occupation, location, places in the world they had experienced living, and typical transport modes.

#### 2.3.3. Scenario Introduction and Instructions

**Figure 10:**
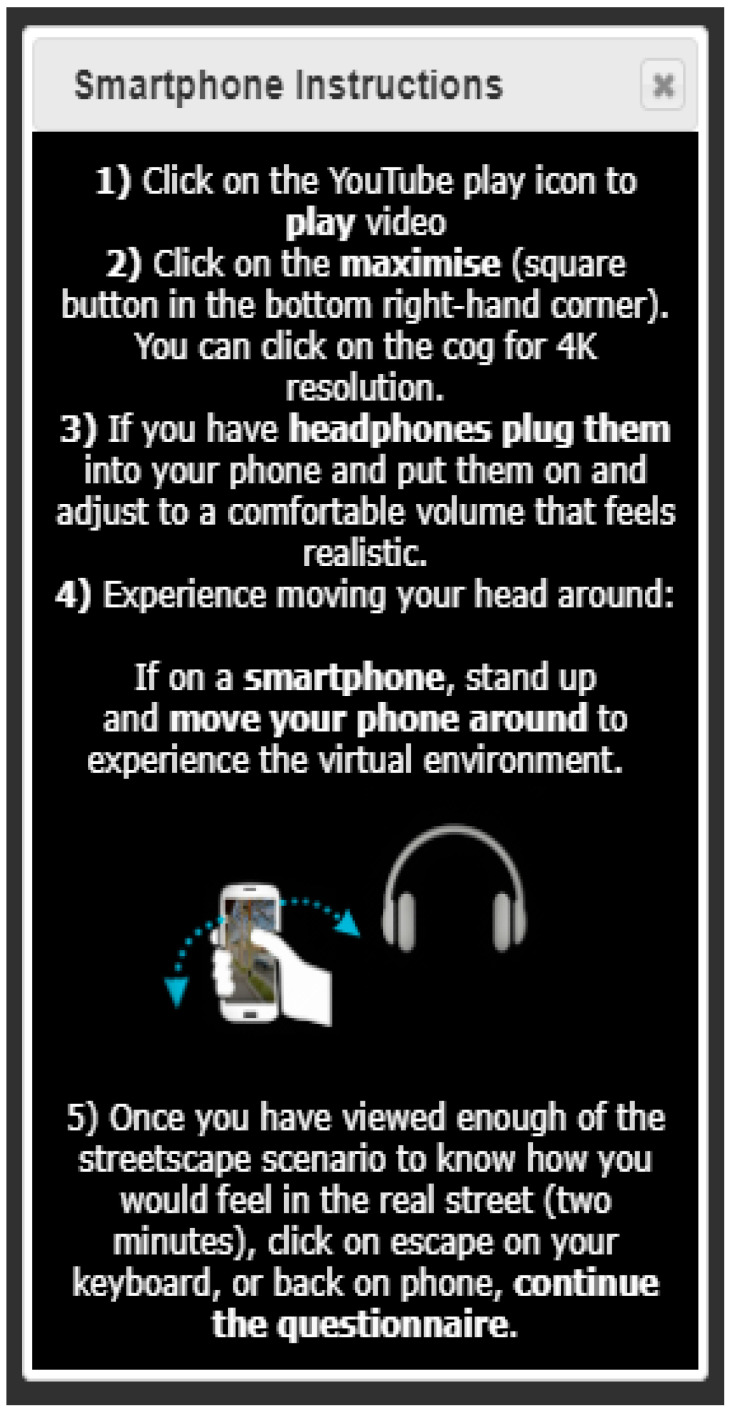
Screen grab of smart phone instructions for viewing and listening to the streetscape scenarios.

**Figure 11:**
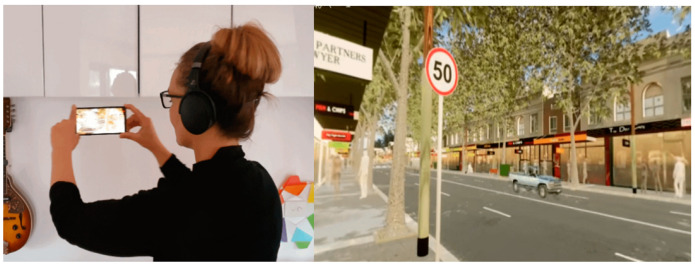
Animated .gif images: (**Left**) showing participant moving their smart device around to see different angles of the immersive virtual environment, and (**Right**) showing a screen capture of what the participant sees on their smart device screen.

Participants were then provided with a simple explanation of how the streetscape scenario questions were to be answered with instructions asking them to 'imagine you are standing on the footpath in the different streetscapes while viewing and listening to the 360-degree videos', and simple instructions on how to use their device with headphones to experience the scenarios [Figure 10]. Then they were told that after viewing the scenario, they would be asked questions about how they felt in these streetscapes, how they would feel about crossing the street, and which elements of the street they thought made the space feel more, or less, pleasant and safe. We provided an illustration of the viewing process using an animated .gif image showing a participant moving their smart device (phone with inbuilt gyroscope) while viewing an example scenario, with an animated .gif image of what was being seen on the phone [Figure 11]. We also provided instructions to describe the process of ‘maximising’ the view of the video to take up the whole screen, and how to ‘move your head’ (direction of view) around to experience the virtual environment.

#### 2.3.4. Randomised Scenarios with Associated Safety and Place Questions

After the participants had moved past the instructions, each of the immersive virtual environment streetscape scenarios was presented as an embedded 360-degree video within the survey. A simple instruction ‘reminder’ was included as a ‘pop-up’ above the video if needed by the participant. To avoid potential order effect bias (where a participant may react differently to questions based on the order in which questions appear), the streetscape scenarios were run in a randomised order via computer-generated sequence.

**Figure 12:**
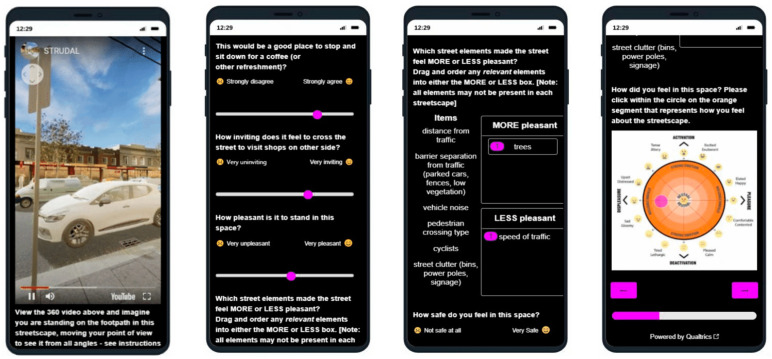
Screen grabs of smart phone interface showing 360-degree immersive virtual environment streetscape scenario embedded into the survey, questions about user experiences using adapted ‘emotion-affect’ sliders and emoji-affect-grid.

After experiencing the immersive virtual environment (2 min), participants were then prompted to answer a series of questions that used an adaptation of the ‘emotion/affect slider’ [28,29] with ‘Visual Analogue Scale’ [30] [Figure 12]. Participants were asked to respond to questions about the quality of the place to stop and sit down for a refreshment, about crossing over to visit shops on the other side of the street, and how pleasant the space felt on a digital analogue scale (slider) with emojis representing simple emotions. Participants were then asked to ‘drag-and-drop’ text items into two different boxes in order to prioritise relevant streetscape design elements, one box representing elements that made space feel MORE pleasant, and one box representing elements that made the space feel LESS pleasant.

Participants were then asked to respond to another emotion affect slider question about how safe they felt in the space, followed by another ‘drag-and-drop’ prioritising question focused on elements that made the space feel MORE safe, or LESS safe [Figure 12].

**Figure 13:**
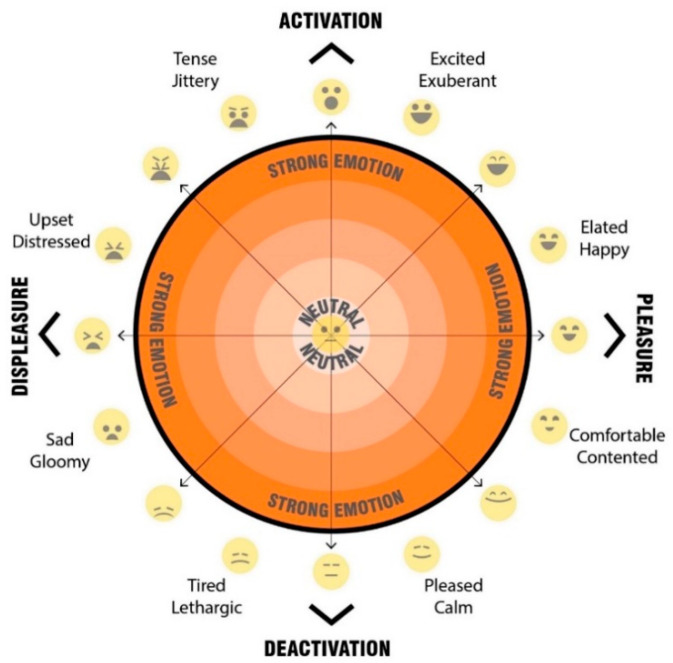
Emoji-affect grid for participants to register their emotional response when asked ‘how do you fell in this space? Please click on the orange segment that represents how you feel about the streetscape’.

The final question for each scenario asked participants to mark their psychological response to the space on a 2D ‘affect grid’ [Figure 13]. The affect grid, based on an adaptation of the method as described by Russell et al. [31] was combined with the EmojiGrid, an emotional representation with the use of emojis for affect pleasure and emotion [32].

Our affect grid was design to allow participants to register their emotional response, by using their mouse, or finger to register a point within the orange circle, to indicated their current emotion/mood. Depending on the location of the point registered, it falls into either the top half—activation (what Russell called ‘arousal’), the bottom half—deactivation (what Russell called ‘sleepiness’), the left hand side—displeasure (unpleasant feelings), and the right hand side—pleasure (pleasant feelings). This provides four quadrants which are then broken down to more nuanced feelings. Levels of intensity (a higher level of the emotion) are registered through distance outward from the neutral centre (darker orange colour).

#### 2.3.5. Post Scenario Questions

After experiencing the streetscape scenarios, participants were asked a set of final questions to collect their overall experience of the streetscapes. They were also asked a series of questions to evaluate the extent to which they felt immersed in the environment and how real it felt based upon the presence in virtual environments questionnaire by Witmer & Singer [33].

### 2.4. Recruitment of Participants

The study aimed to gain generalisable yet actionable insigts into street user preferences removed from specific place based experience or memory, and at greater numbers that ususally achievable at localised community consultation events. Thus we aimed to attract participants from different ages, backgrounds and occupations and locations. The only exclusion from the study was for those minors (under the age of 16 years of age), who were unable to consent to the survey. Recruiting for the study involved the use of a ‘snowball’ approach and was initially shared to the community via media posts, website/blogs, emails, social media (Twitter, FB, Instagram, LinkedIn) and an advertisement on Facebook. To enable a confidence level of 95% (the probability that the sample accurately reflects the attitudes of the population), with a margin of error under 10% (the range the population’s responses may diverge from the sample), we sought a minimum of 100 participants, but as we will discuss in the next section, the response rate was considerably higher than this.

### 2.5. General Estimating Equation (GEE) Ordinal Logistic Regression

Four analysis models were conducted to determine whether the 12 streetscape scenarios had statistically significantly different responses to the ‘Baseline scenario for each question. We chose a general estimating equation (GEE) method with an ordinal logistic regression as the chosen model. We chose this model because GEE is useful in handling repeated measures studies and ordinal logistic regression handles Likert scale data well.

## 3. Results

In this section, will provide a summary of the results of the survey relating to the effectiveness of the e-participation method in eliciting responses from citizens. To understand the validity of the study, here we will explore the volume of responses and the answers to some important demographic questions including the breakdown of genders, ages, occupation, and location, as well as to know the common modes of travel of participants. While the detailed exploration of the results of stated and revealed perception and preference questions is beyond the scope of this paper, we will provide samples of the kinds of data analytics that the survey provided.

### 3.1. Responses

#### 3.1.1. Response Rate

The response rate to the survey was greater than anticipated with over 900 participants, of which there were over 276 valid responses. This rate of completion and valid responses were considerably higher than expected, given the length of the survey and the level of detailed responses required. This sample size provides results with a confidence level of 95%, and a margin of error of 6%.

#### 3.1.2. Gender Breakdown

**Figure 14:**
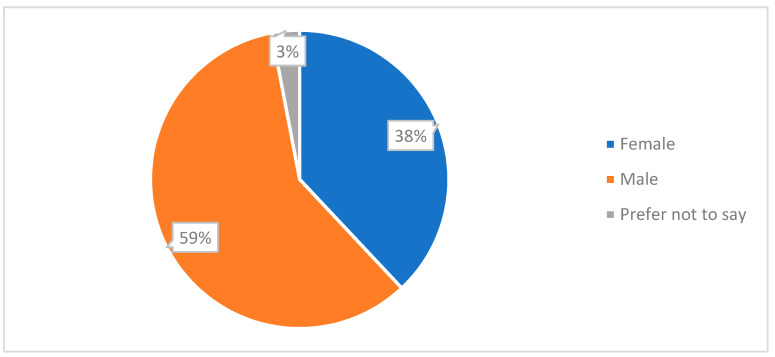
Pie chart showing gender distribution.

Responses to the survey had a gender distribution of 59% of participants identified as male, 38% of participants identified as female, and the remainder were identified as other or preferred not to say [Figure 14]. Responses were analysed for any impact gender may have had on results. We found no significant differences in responses between males and females.

#### 3.1.3. Location of Participants

We found that the location of the survey participants, according to postcode, were predominantly from NSW with just under two thirds (63.9%) of the responses. The remaining participants included just under one fifth from Victoria (19.7%), and small numbers from WA, ACT, QLD, SA and TAS. Participants within NSW were generally located along the East Coast with a concentration within Sydney. There was a reasonable spread of participants across the wider metropolitan, and a concentration found in the areas closer to, and within central Sydney.

#### 3.1.4. Age of Participants

**Figure 15:**
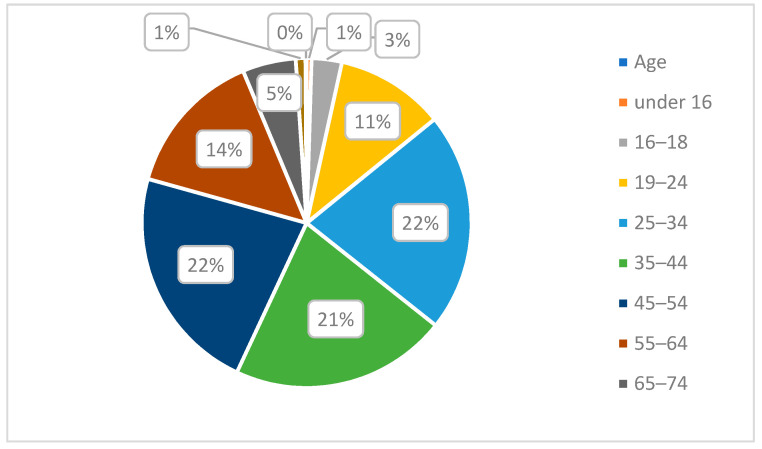
Pie chart showing the distribution of stated age categories of participants.

When asked to choose from the series of age categories, we found participant response to have a reasonable spread across each of age groups with the exception of the 75–84-year-old (1%) and the over 85 category (less than 1%) [see Figure 15], which was expected as a consequence of a survey limited to an online-only format. A small percentage of responses was found in the 16–18 senior-school age group (3%), but as this is a smaller age range (just two years), the percentage was considered relatively even with other age categories. When response to key questions were analysed against age category, we found that opinions tended to be more positive, the younger the participant, with participants under the age of 34 were on average more likely to responded positively in all scenarios.

#### 3.1.5. Occupation of Participants

**Figure 16:**
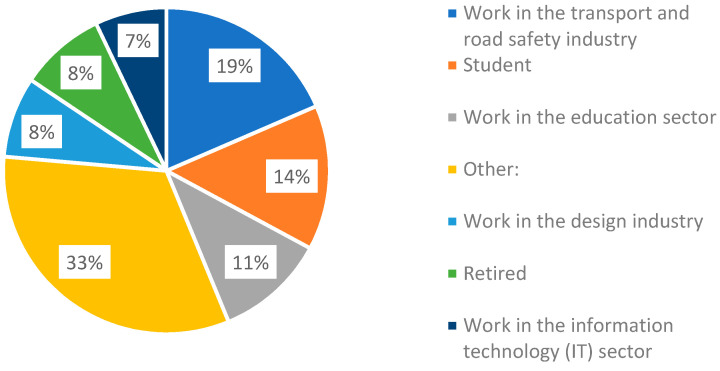
Pie graph showing the percentage breakdown of respondent’s response to the question of occupation.

The question of occupation was included in the survey to enable checking for any potential bias relating to the occupation. We found a high level of participation from those stating they worked in industries related to transport, and education, as well as students [Figure 16]. We also found that participants in the study represented a diverse range of occupations including the health sector, engineering, finance, hospitality and, defense, through to a tailor and a zookeeper.

We did not find significant differences between answers to the four key affect slider questions, across all scenarios by occupation field, other than the question: ‘Would this be a good place to stop for coffee?’, to which designers responded 54% more positively than all other occupations [Exp(B) 1.544]. When we compared the transport field to all other occupation fields as a combined group, we found there were no significant differences between these two groups for any of the questions. Some of the significant levels were close to 1, indicating almost no difference between those who work in transport and those who work in other fields.

For the specific scenarios where the research team believed there may potentially be an occupation-based bias in respondents who worked in transport due to their professional training: the ‘Parked car buffer’ scenario, and the ‘Barrier fence’ scenario, we compared the responses of those in the transport field with all other occupations combined as a single group. Between these two groups, there were no statistically significant differences for any of the questions for those two selected scenarios.

#### 3.1.6. Transport Modes

**Figure 17:**
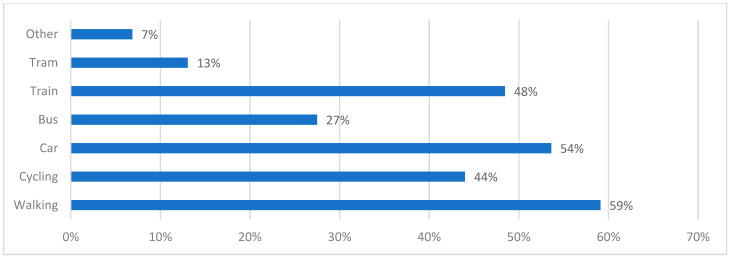
Nominated modes of transport commonly used by participants. Participants were permitted to choose multiple transport modes.

Respondents were asked to nominate transport modes they commonly used from a list of seven (including ‘other’). Respondents were allowed to choose multiple transport modes [Figure 17]. Across all scenarios, for all questions, the impact of transport mode was minimal, though there were small statistically significant differences in the responses of those who selected trams and those who selected cycling, both of whom were more likely to be negative than those who did not select trams or cycling. This was true in all four questions apart from: ‘How safe do you feel in this space?', for which there was no statistically significant difference between those who did and those who did not select trams as a common transport mode. As none of the scenarios included trams in the street, the slightly lower number of positive responses from those who selected trams is not unexpected.

Non-cyclists were on average 45.6% more likely to have positive responses compared to cyclists to the question: Would this be a good place to stop for coffee? (*p* < 0.001). Non-cyclists were on average 39.1% more likely to have positive responses to the question: How inviting does it feel to cross the other side? (*p* = 0.002). They were on average 45.9% more likely to have positive responses to the question: How pleasant is it to stand in this space? (*p* < 0.001), and 30.3% more likely to have positive responses to the question: How safe do you feel in this space? (*p* = 0.009).

### 3.2. Sample Survey Response

#### 3.2.1. Sample of Revealed Preference Affect-Slider Results

The study provided a wealth of data that allows for the detailed interrogation of citizen perceptions of a variety of streetscape treatments with statistically significant results. In this section of the paper, we will give a sample of the kind of insights gained through the application of the ordinal logistic regression analysis using GEE for analysing the responses to the survey question ‘Would this be a good place to stop for coffee (or other refreshments)?’. Compared to the ‘Baseline’ scenario, statistically significant responses to 8 of the 12 scenarios were more positive. The ‘Cycle lane’ (*p* < 0.001) responses were on average 3.97-times more likely (3.01, 5.14) to be positive, the ‘Wombat crossing’ (*p* < 0.001) responses were on average 3.48-times more likely (2.44, 4.99) to be positive, the ‘Reduced speed 30 km/ph’ responses were on average 3.43-times more likely (2.69, 4.37) to be positive, the ‘Refuge island’ (*p* < 0.001) responses were on average 3.12 (2.19, 4.44)-times more likely to be positive, the ‘Widened footpath’ (*p* < 0.001) responses were on average 2.43-times more likely (1.91, 3.08) to be positive, the ‘Parked car buffer’ (*p* < 0.001) responses were on average 2.36-times more likely (1.49, 3.72) to be positive, the ‘Increased tree canopy’ (*p* < 0.001) responses were on average 72.9% more likely (1.397, 2.140) to be positive and the ‘Barrier fence’ (*p* < 0.001) responses were on average 59.3% more likely (1.245, 2.039) to be positive. The results for the scenarios ‘Ground vegetation buffer’, ‘Sig. cross long-wait’, ‘Sig cross short-wait’ and ‘Less clutter’ did not significantly differ from the ‘Baseline’ scenario. These results are summarised in Table 2.

#### 3.2.2. Sample of Revealed Preference Affect-Grid Results

**Figure 18:**
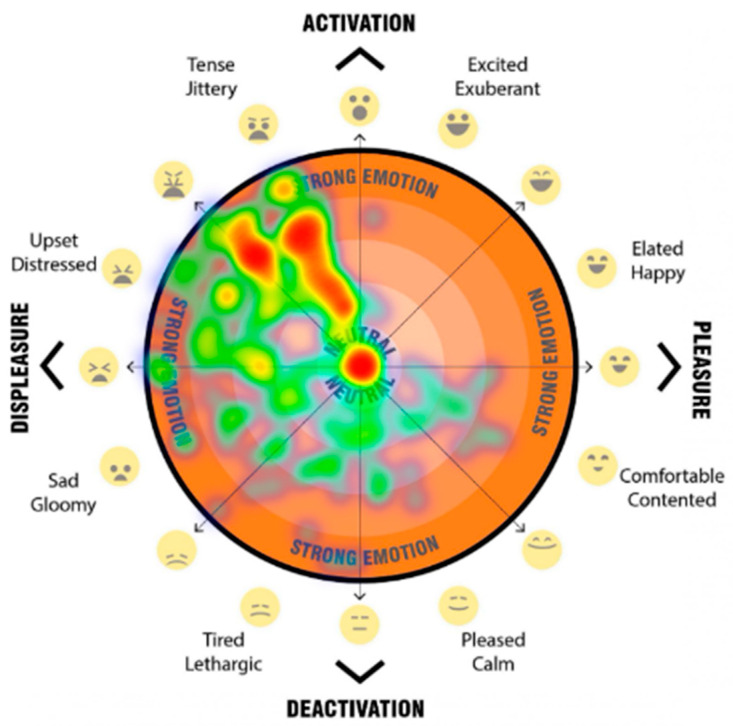
Heatmap visualises the ‘Baseline’ scenario responses of the affect grid for participants to register their emotional response when asked ‘How do you feel in this space?’

**Figure 19:**
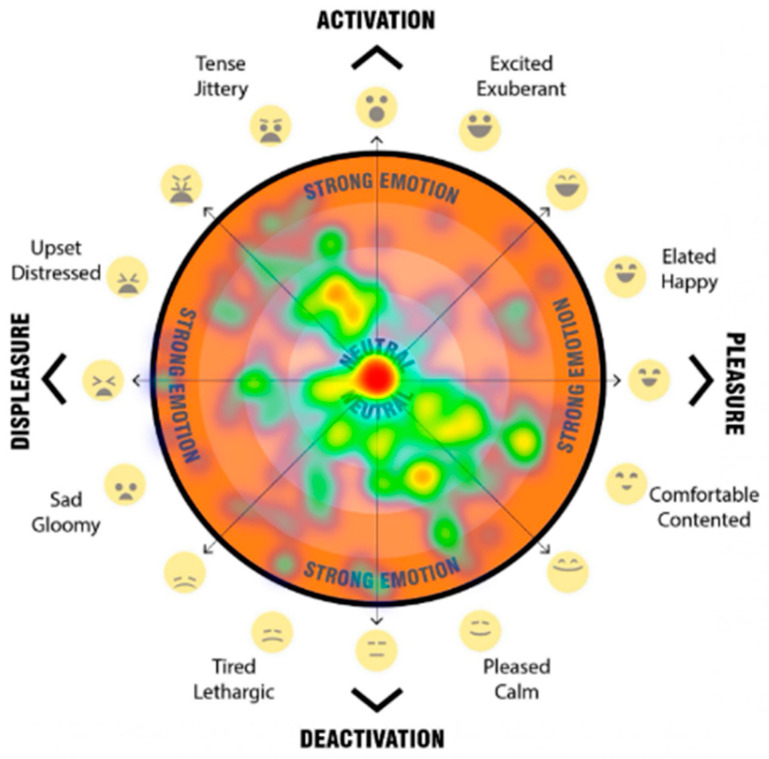
Heatmap visualises the Reduced speed (30 km/ph) scenario responses of the affect grid for participants to register their emotional response when asked ‘How do you feel in this space?’

**Figure 20:**
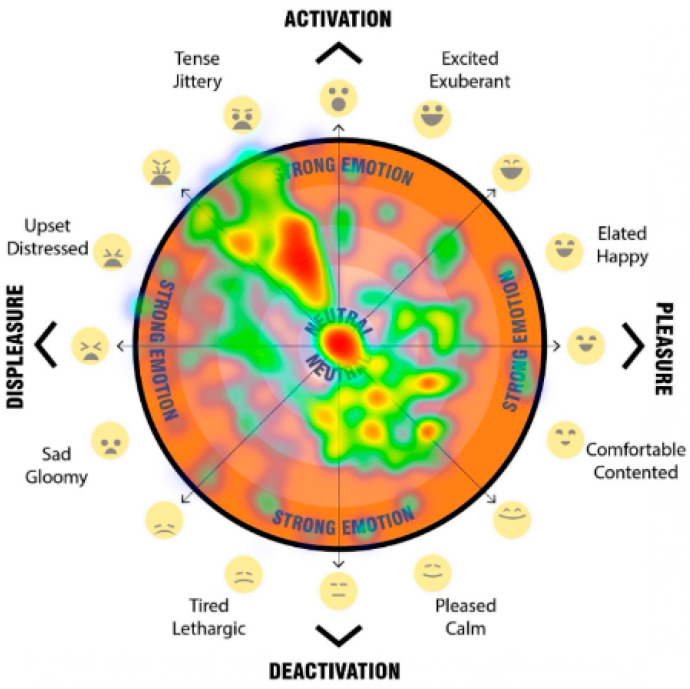
Heatmap visualises the increased tree canopy scenario responses of the affect grid for participants to register their emotional response when asked ‘How do you feel in this space?’

**Figure 21:**
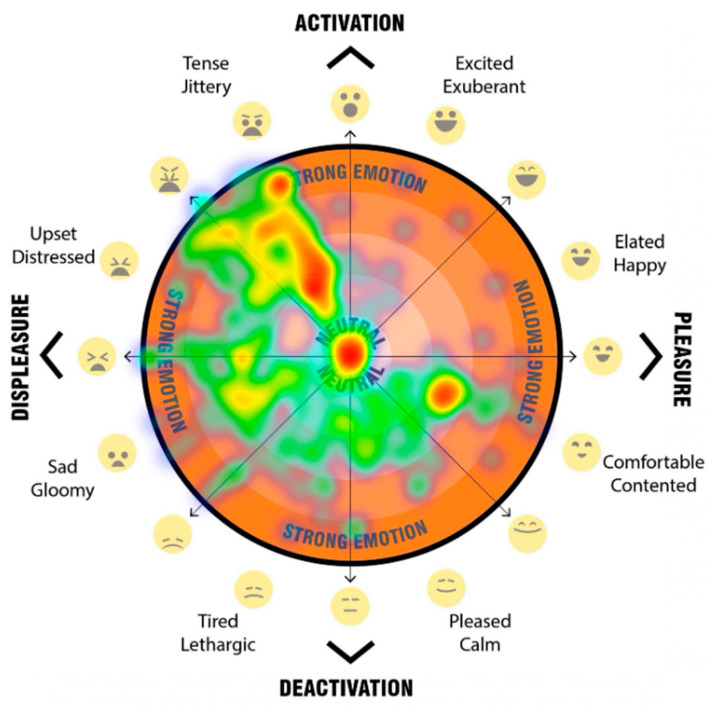
Heatmap visualises the Barrier fence scenario responses of the affect grid for participants to register their emotional response when asked ‘How do you feel in this space?’

The participant’s responses when participants were asked to register their emotional response when asked ‘How do you feel in this space? Please click on the orange segment that represents how you feel about the streetscape’ were registered on the emoji affect grid [Figure 13]. Participants used their mouse or finger to register a point upon the affect grid, which indicated their emotional response to each streetscape scenario. Depending on the location of the point registered, we captured the response and categorised the point as falling into either the top half—activation, the bottom half—deactivation, the left-hand side—displeasure, and the right-hand side—pleasure. We also categorised the response data based on the overlapping halves as quadrants (activation-displeasure, activation-pleasure, deactivation-displeasure and deactivation-pleasure). In addition, we analysed the more nuanced feelings and levels of intensity based on the registered response point location where responses marked further away from the neutral centre (darker orange colour), the higher the level of emotion. The detailed analysis of these results are beyond the scope of this paper, but a sample of the visual comparisons of the impact on feelings of pleasure, displeasure and level of activation is shown in Figure 18, Figure 19, Figure 20 and Figure 21. In these examples, we can see a clear positive shift in affect from responses from the ‘Baseline’ scenario [Figure 18] which showed almost no responses in the activated and pleasurable quadrant, to ‘Reduced speed’ [Figure 19], ‘Increased Tree Canopy’ [Figure 20] and ‘Barrier Fence’ [Figure 21] scenarios which all show higher spread into either the active pleasure, ore calmer (deactivated) pleasure quadrants.

#### 3.2.3. Sample of Stated Preference

When ranking the elements that made the street feel more pleasant, across all scenarios the three most popular elements to rank no. 01 for making a street more pleasant were first trees, then separation from traffic and thirdly the presence of cyclists. Trees were the most popular choice to be ranked no.1 for elements that make a street feel more pleasant, consistent across all scenarios. Separation from traffic (through parked car buffers, fences, ground vegetation, wider footpaths and cycle lanes), was the second most popular choice to be ranked no.1 for elements that make a street feel more pleasant. Responses to two of the three scenarios that increased the distance separation between pedestrians and moving traffic (‘Wider footpath’ and ‘Cycle lane’), distance separation was in the top three most popular elements to rank no.01 for making those scenarios MORE pleasant. The presence of cyclists was the third most popular choice to be ranked no.1 for elements that make a street feel more pleasant. In the four crossings scenarios, crossings were either the second or third most popular choice of element that make a street feel more pleasant.

## 4. Discussion

### 4.1. Key Methodological Insights

This study combined multiple novel methods for representing streetscape elements and measuring participant responses. Key methodological insights include the following.

#### 4.1.1. The Approach Garnered a Strong Level of Engagement

The digital survey format and content appear to have been suitably engaging with the number of valid response-rates being considerably greater than expected (over 900), with nearly one third mid-way to full completion of survey. The high numbers of responses allowed for eliciting large numbers of statistically significant results. Participants were also found to be spread across the wider Sydney metropolitan area as well as throughout the state of NSW.

#### 4.1.2. The Digital Survey (E-participation) Approach Resulted in Input from a Wide Range of Citizens

By using the e-participation approach to the method for engagement, it allowed us to reach a broad sector of society. Citizens were not required to be available to physically attend workshops, negating some of the issues usually associated with streetscape change consultation forums which can be restricted to highly-localised groups and individuals [34]. While the e-participation approach did appear to somewhat limit engagement from older adults, in a post-pandemic environment, it would be possible to augment the study with assisted in-person e-participation.

#### 4.1.3. Using Immersive Virtual Environments (IVE) Allowed for Multiple Controlled Scenario Analysis

Using immersive virtual environments (IVE) allowed for controlled variable adjustments, enabling us to test many different scenarios consecutively, while overcoming limitations of ‘recall’ methods that rely on a participant’s memories of real-world spaces. This IVE approach improves on previous methods such as use of photographs, modified photographs and videos, overcoming difficulties in providing constant environmental conditions for fair comparison as described Ewing and Handy, and Moudon and Lee [9].

#### 4.1.4. The IVEs Allowed for the Testing of Complex Dynamic Street Interactions

Modelling the complex interaction of streetscape systems and space to produce clear, realistic, dynamic and multi-sensory stimuli with minimal confounding variables, overcomes limitations of still photographic image comparison methods. The IVE method using a de-identified streetscape (based on a real street), also negated biases associated with personal experiences of specific local spaces.

#### 4.1.5. The Approach Allowed for the Quantification of Human Emotional and Perceptual Responses

The use of this e-participation method, with its inclusion of interactive emotion sliders and the affect grid allowed us to quantify human emotional and perceptual responses to the streetscape scenarios [Figure 18, Figure 19, Figure 20 and Figure 21]. The approach also allowed us to understand which interventions on streets have the most positive or negative impacts on perceptions of safety and place. These results show the potential for quantifying human response to different urban environments and begin to objectively measure subjective qualities of the urban street environment, what Ewing and Handy call ‘measuring the unmeasurable’ [8].

### 4.2. Limitations

Due to the impact of COVID-19, this study was to online-only and required using participant’s own devices and headphones introducing limitations in terms of environmental and audio consistency across the study. As each participant was completing the survey online, we could not control the environment where the survey was conducted. They may have been in agitating environments for example, which may skew their responses. Another limitation was that the researchers were not able to see participants as they were using the immersive virtual environment, trouble shoot if the participant was having any problems or clarify any questions.

This study was aimed at engagement with the population as a whole, and not specifically aimed at gauging different attitudes between age groups. For a more detailed analysis on different perceptions of safety and place for different age groups, ideally, we would have more people do it from each different age group, particularly participants younger than 16 years old who were omitted from the study (due to complexities in obtaining parental consent), and more responses from the population of older adults, people aged 75 and above). In a post-COVID environment, we hope to continue this study with an in-person option to allow for these under-represented age groups to participate fully. The survey format may not have been suitable or fully accessible, specifically for people with a visual impairment who may have found the survey visual content and response methods difficult to complete.

The survey recruitment was not entirely random. Like most studies of this kind, people have self-selected to agree to fill in the survey, and though it appears that we have managed to elicit good representation across genders, cultural backgrounds, occupations and ages, participants were all within sectors that were either interested in streetscape design, interested in immersive virtual environments, or were willing to commit 20–30 min to the study.

There is also a good representation of people who use different transport modes—with 44% of people identifying as people who use cycling as a ‘form of transport’. Data from both the City of Sydney and City of Melbourne suggest that cycling as a mode of commuting (daily travel to and from work) is relatively low for the two cities (under 10%). Cycling as a ‘form of transport’ and ‘for commuting’ are clearly not the same, and we would expect to see this higher number of people using cycling as one component of their more general ‘forms of transport’ options, but these results were interesting and warrant further research.

Streetscapes are highly complex, adaptive systems with hundreds of interwoven variables. While we have attempted to simplify and limit our scenario variations to changing ‘single variables’ where possible, it was not possible to isolate a single variable for each scenario. This complexity meant that for some scenarios, it is not possible to completely isolate the element that is impacting on the participant’s safety and place.

Due to project constraints, as well as the issue of survey fatigue, the number of scenarios we could test was limited. There were many important and worthy variables that were not included in this initial pilot study due to this limitation. We also limited the study to a consistent ‘typical’ street section on a ‘main street’ typology. Other street widths and different levels of building enclosure (height to width ratios) may have a significant impact on streetscape perceptions, as may different densities of populations and street crowding.

### 4.3. Suggested Further Research—What Next?

While this project has demonstrated the successful application of a new e-participation approach that combines online survey methods with 4D animated immersive virtual environments, an affect grid, and emotion response sliders to quantify and assess citizen’s perceptions of safety and place, results and insights gained from the project suggest great potential in expanding on this research. Further work is recommended to build on the outcomes of this project., the following in particular: as limitations on in-person engagement and COVID19 related restrictions are lifted, the study could be extended to include in-person assisted e-participation that would be more inclusive for older adults who were underrepresented in the study; further refinement of the online-survey interface could also improve accessibility for people with visual impairment; the impact of different devices, resolutions and audio experiences (headphones) could be further explored through a series of controlled environment experiments; the level of immersion in the streetscape environments could also be explored by comparing full virtual reality with Head Mounted Displays against the immersive 360-degree videos in this study; and the stakeholder workshop raised a wide range of interesting streetscape variables and possible scenarios that were outside of the project scope, many of which would be worth exploring using the same methodology.

There are also several potential areas of interest into which this work could expand, such as: an exploration of potential impacts of COVID-normal NYC ‘open restaurant’ expansion of retail onto the roadway; treatments for other street types could be explored including Local Streets, Main Roads, and Civic Places, investigation of the impact of different tree species and spacing, on perceptions of place; the potential impact of electric vehicles (given the reduction in engine noise) on perceptions of place; to explore the impact of various ‘micro-mobility’ vehicles such as e-bikes, e-scooters and their parking on place; and to explore the future impact of Autonomous Vehicles and significant potential street reconfigurations.

## 5. Conclusions

Using embedded Immersive Virtual Environments (IVE) as visual and auditory stimulus within an e-survey tool coupled with three types of questions—revealed preference (gathered through rating scale questions), stated preference (gathered through element ranking) and emotion affect (gathered through affect grid questions) is a robust method for understanding human preferences and perceptions of safety and place of individual variables of streetscapes with input from a large numbers of participants. The use of IVE enabled two important innovations. Firstly, it focused responses from a forced point of view (POV), that of the pedestrian, regardless of respondent’s personal day-to-day transport choices, and secondly, through virtue of being simulated, reduced confounding emotional responses based on personal experience of specific streets. The use of an online survey enabled the gathering of data from a larger user group with a broader geographic range than is possible using traditional community consultation methods that focus on smaller, localised stakeholder groups.

The method outlined in this paper has great potential to become part of the design process for transport authorities, urban designers and local governments. The outcomes of this study can be used to inform development and planning in both existing and greenfield areas of high-quality successful places that allow for movement, feel pleasant, are vibrant, are comfortable stopping places, and importantly, feel safe.

## Figures and Tables

**Figure 3:**
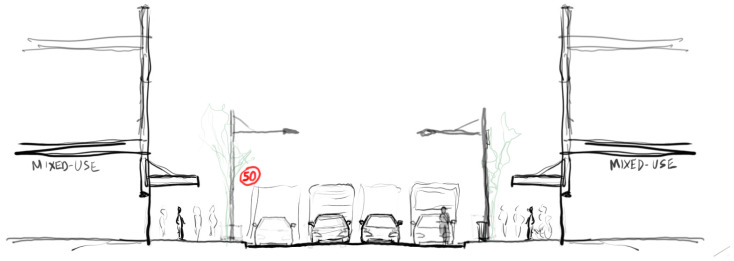
Example of streetscape scenario, sketch drawing of street section showing the ‘Baseline’ scenario.

**Figure 4:**
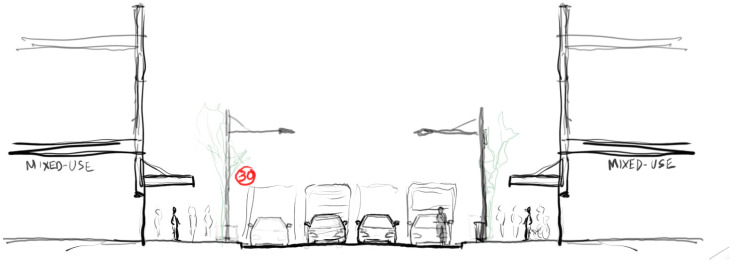
Street section sketch showing the ‘Reduced speed’ (30 km/h) scenario.

**Figure 5:**
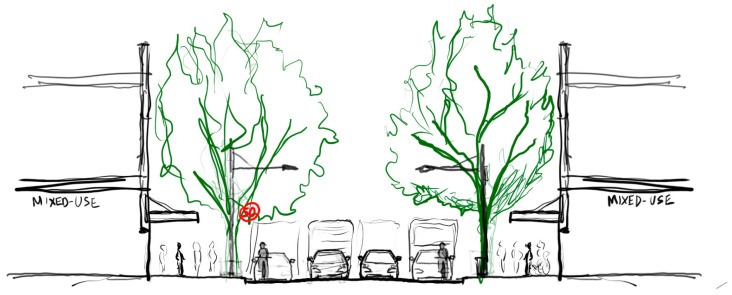
Street section sketch showing the ‘Increased tree canopy’ scenario.

**Figure 6:**
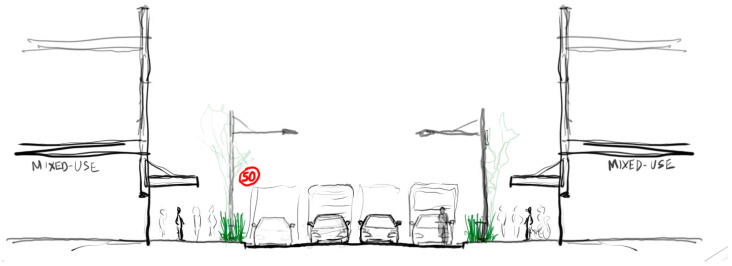
Street section sketch showing the ‘Ground vegetation buffer’ scenario.

**Figure 7:**
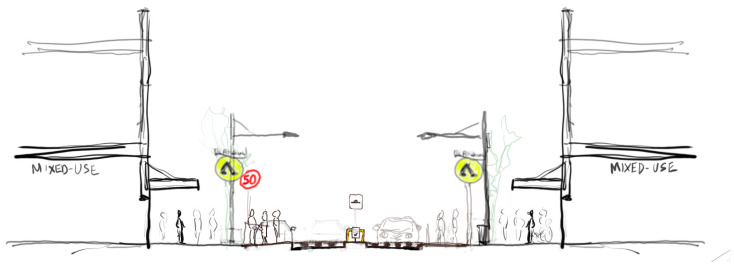
Street section sketch showing the ‘Wombat crossing’ scenario.

**Table 1:** Table of the final streetscapes chosen to be modelled including a single baseline and 12 street variations.

Prioritised Streetscape Table
Baseline control configuration: ‘Baseline’ (4 lanes of traffic @50 km/ph) [Figure 3]
Speed configuration: ‘Reduced speed’ 30 km/ph [Figure 4]
Distance separation configuration: ‘Parked car buffer’ (2 lanes of traffic)
Barrier separation configuration: ‘Barrier fence’
Barrier separation configuration: ‘Increased tree canopy’ [Figure 5]
Barrier separation configuration: ‘Ground vegetation buffer’ [Figure 6]
Distance separation configuration: ‘Cycle lane’ (3 lanes of traffic)
Barrier separation configuration: ‘Less clutter’
Distance separation configuration: ‘Widened footpath’ (3 lanes of traffic)
Crossing configuration: ‘Signalised crossing long-wait’ (non-pedestrian prioritised)
Crossing configuration: ‘Signalised crossing short-wait’ (pedestrian prioritised)
Crossing configuration: ‘Refuge island’ (non-pedestrian prioritised) (2 lanes of traffic)
Crossing configuration: ‘Wombat crossing’ (pedestrian prioritised) (2 lanes of traffic) [Figure 7]

**Table 2:** Statistically significant (*p* < 0.001) findings ranked for ‘would this be a good place to stop for coffee?’

Ranked Findings for ‘Would This Be a Good Place to Stop for Coffee?’
‘Cycle lane’ (3.97-times more likely to be positive) ‘Wombat crossing’ (3.48-times more likely to be positive) ‘Reduced speed’ 30 km/ph (3.43-times more likely to be positive) ‘Refuge island’ (3.12-times more likely to be positive) ‘Wider footpath’ (2.43-times more likely to be positive) ‘Parked car buffer’ (2.36-times more likely to be positive) ‘Increased tree canopy’ (72.9% more likely to be positive) ‘Barrier fence’ (59.3% more likely to be positive)

## Data Availability

Additional data for this project can be found at https://imoveaustralia.com/project/project-outcomes/exploring-balance-between-movement-and-place-in-designing-safe-and-successful-places/ (accessed on 1 January 2023).
